# Targeting BCL-2 in B-cell malignancies and overcoming therapeutic resistance

**DOI:** 10.1038/s41419-020-03144-y

**Published:** 2020-11-02

**Authors:** Isha Kapoor, Juraj Bodo, Brian T. Hill, Eric D. Hsi, Alexandru Almasan

**Affiliations:** 1 Department of Cancer Biology, Lerner Research Institute, Cleveland, OH USA; 2 Department of Laboratory Medicine, Institute of Pathology and Laboratory Medicine, Cleveland, OH USA; 3 Department of Hematology and Medical Oncology, Taussig Cancer Institute, Cleveland, OH USA; 4 Department of Radiation Oncology, Taussig Cancer Institute, Cleveland Clinic, Cleveland, OH 44195 USA; 5 Case Western Reserve University School of Medicine, Cleveland, OH 44106 USA

**Keywords:** Chronic lymphocytic leukaemia, Chronic lymphocytic leukaemia

## Abstract

Defects in apoptosis can promote tumorigenesis and impair responses of malignant B cells to chemotherapeutics. Members of the B-cell leukemia/lymphoma-2 (BCL-2) family of proteins are key regulators of the intrinsic, mitochondrial apoptotic pathway. Overexpression of antiapoptotic BCL-2 family proteins is associated with treatment resistance and poor prognosis. Thus, inhibition of BCL-2 family proteins is a rational therapeutic option for malignancies that are dependent on antiapoptotic BCL-2 family proteins. Venetoclax (ABT-199, GDC-0199) is a highly selective BCL-2 inhibitor that represents the first approved agent of this class and is currently widely used in the treatment of chronic lymphocytic leukemia (CLL) as well as acute myeloid leukemia (AML). Despite impressive clinical activity, venetoclax monotherapy for a prolonged duration can lead to drug resistance or loss of dependence on the targeted protein. In this review, we provide an overview of the mechanism of action of BCL-2 inhibition and the role of this approach in the current treatment paradigm of B-cell malignancies. We summarize the drivers of de novo and acquired resistance to venetoclax that are closely associated with complex clonal shifts, interplay of expression and interactions of BCL-2 family members, transcriptional regulators, and metabolic modulators. We also examine how tumors initially resistant to venetoclax become responsive to it following prior therapies. Here, we summarize preclinical data providing a rationale for efficacious combination strategies of venetoclax to overcome therapeutic resistance by a targeted approach directed against alternative antiapoptotic BCL-2 family proteins (MCL-1, BCL-xL), compensatory prosurvival pathways, epigenetic modifiers, and dysregulated cellular metabolism/energetics for durable clinical remissions.

## Facts

1. BCL-2 family proteins are key regulators of the intrinsic, mitochondrial apoptotic pathway.
2. Therapeutics that target BCL-2 show significant clinical activity for the treatment of B-cell malignancies.
3. Cancer cells evade apoptosis by acquiring resistance to BCL-2 inhibition and oncogenic stress.
4. Metabolic adaptation and dysregulated cellular energetics likely contribute to resistance to BCL-2 inhibition.

## Open questions

1. How do prior therapies enable or potentiate the efficacy of BCL-2 family inhibitors?
2. How can we make resistant tumors to become dependent on BCL-2?
3. Is targeting deregulated metabolism a selective approach to inhibit cancer cells and overcome drug resistance?
4. What are the optimal drugs to combine with BH3 mimetics for maximum efficacy in different cancers?

## Introduction

Lymphoid malignancies account for the majority of hematological neoplasms. It has been estimated that ~86,400 new cases of non-Hodgkin B-cell lymphoma (NHL), including ~20,980 cases of chronic lymphocytic leukemia (CLL)/ small lymphocytic lymphoma (SLL) were diagnosed in the US in 2016^1^. The highest incidence among these has diffuse large B-cell lymphomas (DLBCL), followed by CLL/SLL and follicular lymphomas (FL)^1^. Impaired apoptosis and clonal proliferation of lymphocytes at different stages of maturation is a cardinal feature of many cancers, including NHL^2^ and CLL^3,4^. NHL has historically been treated with either monoclonal antibodies alone or in conventional chemotherapeutic agents with more recent introduction of specific signaling pathway inhibitors for some NHL subtypes. Overall, survival varies significantly in different subtypes and stages of the disease^2^.

**Fig. 1:**
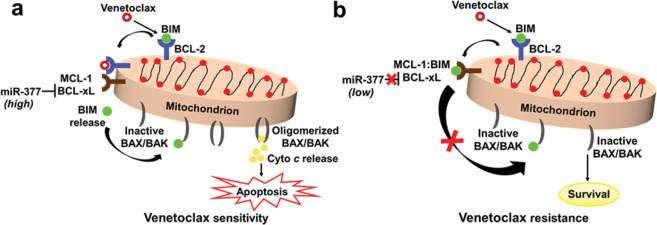
Mimicking the BH3 domain to kill tumor cells. **a** The BH3-mimetic venetoclax targets the antiapoptotic BCL-2 protein in sensitive cells and displaces proapoptotic BIM from BCL-2 to cause activation of effector proteins BAX or BAK leading to apoptosis via cytochrome *c* release. **b** As venetoclax does not target antiapoptotic MCL-1 and BCL-xL proteins, these proteins confer resistance via sequestration of BIM displaced from BCL-2. Epigenetic miR-377 downregulation by methylation results in increased expression of BCL-xL in resistant cells.

The mitochondrial pathway of apoptosis is governed by the B-cell lymphoma 2 (BCL-2) family of pro- and antiapoptotic proteins. Across B-cell malignancies, apoptosis dysregulation can result from overexpression of the BCL-2 protein that can sequester certain proapoptotic BCL-2 homology 3 (BH3)-only proteins (e.g. BIM, BID) to prevent oligomerization of pore-forming proteins (BAX and BAK) and subsequent mitochondrial outer membrane permeabilization (MOMP) (Fig. 1)^5^. Within B-cell tumors, BCL-2 dysregulation commonly arises from genetic abnormalities, such as the *t*(14;18)(q32;q21) translocation in follicular lymphoma (FL) or focal deletion of chromosome 13 (*del*[13q14]) that leads to loss of negative regulatory miRNA-15a/16-1 of BCL-2 in CLL. Thus, BCL-2 has been a rational therapeutic target in lymphoid cancers^3^.

Venetoclax (ABT-199)^6^ is the first FDA-approved highly selective oral BCL-2 antagonist for the treatment of CLL. It displaces proapoptotic BH3-only proteins from BCL-2, facilitating activation of proapoptotic BAX or BAK proteins to induce apoptosis (Fig. 1a). As monotherapy and in combination with anti-CD20 monoclonal antibodies, it is effective for treating CLL patients including those with high-risk 17p deletion and/or TP53-deficient disease, with high rates of durable complete responses^7–9^. The phase1b CAVALLI study demonstrated impressive clinical outcomes for the combinations of venetoclax with anti-CD20 monoclonal antibodies, rituximab or obinutuzumab (R- or G-CHOP) in NHL^10^. Ongoing randomized clinical trials are testing the addition of venetoclax to standard frontline therapy for patients with DLBCL with Myc and BCL-2 overexpression (NCT03984448). Nevertheless, despite potent clinical activity, recurrence can be observed after a period of initial response, displaying a range of evolutionary patterns. Thus, disease progression is an emerging therapeutic challenge. Such secondary resistance is also observed in other cancers, including NHL and multiple myeloma (MM)^11^.

In this review, we summarize the drivers of de novo and acquired resistance to venetoclax closely associated with complex clonal shifts, interplay of BCL-2 family members, transcriptional regulators, epigenetic modifiers and metabolic modulators. We also examine how tumors initially resistant to venetoclax can become responsive to it following prior therapies. Finally, we summarize preclinical data providing a rationale for strategic therapeutic combinations of venetoclax to maximize therapeutic efficacy and overcome resistance to BCL-2 inhibition in B-cell lymphoid malignancies.

## BCL-2 family proteins, guardians of cell survival

Diverse cytotoxic stimuli, including genotoxic stress, chemotherapeutic agents, as well as developmental cues, engage the mitochondrial pathway to induce MOMP. There are over two dozen members of the BCL-2 family, divided into three functionally and structurally distinct subgroups: the proapoptotic BH3-only proteins, antiapoptotic BCL-2 proteins, and the proapoptotic BCL-2 effector proteins BAX and BAK that are key regulators of MOMP^12,13^. These stimuli initiate MOMP by the activation of BH3-only proteins by post-translational modifications (e.g., caspase 8-mediated cleavage of BID to generate the active truncated form tBID) or transcriptional upregulation (e.g., BIM). Consequently, allosteric conformational changes in BAX/BAK and oligomerization into large multimeric pores in the outer mitochondrial membrane lead to membrane permeabilization and release of apoptogenic factors, such as cytochrome (cyto) *c* into the cytoplasm^12,14^. Assembly and activation of the apoptosome then leads to activation of a caspase cascade and a myriad of effector caspase targets. One such target in hematopoietic tumor cells is Cyclin E. A C-terminal fragment generated by caspase 3-mediated proteolytic cleavage is unable to interact with cyclin-dependent kinase (CDK)2, but instead associates with Ku70, releasing BAX to induce apoptosis^15,16^.

There are two distinct stages of cyto *c* release from mitochondria. The early release of low levels of cyto *c* into the cytosol precedes caspase-9 and -3 activation without affecting ATP levels or mitochondrial membrane potential (MMP). The late stage of cyto *c* release results in MOMP, leading to a drastic loss of mitochondrial cyto *c* and mitochondrial dysfunction, manifested by reduced ATP levels and loss of MMP. This positive feedback amplification loop linking caspase activation to mitochondrial dysfunction could be facilitated by cleavage of antiapoptotic proteins, such as BCL-2, leading to their conversion to a proapoptotic BAX-like molecule^17,18^. Notably, caspase-3-mediated cleavage of the loop domain of BCL-2 activates the latent proapoptotic activity of the BH3 domain of BCL-2 by generating the C-terminal BCL-2 cleavage product that triggers cell death and accelerates apoptosis^18^. The voltage-dependent anion channel associates with BCL-2 family proteins and hexokinase and plays a key role in mitochondria-mediated apoptosis, since after its oligomerization it facilitates the release of apoptogenic proteins located in the mitochondrial inter-membrane space^19^.

Close interactions between these competing BCL-2 family proteins determine the cell fate via an intricate balance between antiapoptotic and proapoptotic proteins to either prevent or initiate apoptosis. BCL-2 and related antiapoptotic proteins, including BCL-xL and MCL-1, inhibit MOMP by binding to and sequestering proapoptotic family members (BIM, BAD, BID, NOXA, and PUMA) indispensable for MOMP and activation of BAK and BAX (Fig. 1b). The link between overexpression of these antiapoptotic BCL-2 proteins and B-cell lymphoid malignancies is now well established, as they underlie clonal proliferation and accumulation of mature clonal neoplastic B cells in the hematopoietic niche leading to failure of molecular targeted therapies^20,21^.

## BCL-2 expression defines tumor dependence and vulnerability

BCL-2 is aberrantly expressed in CLL, FL, mantle cell lymphoma (MCL), Waldenstrom macroglobulinemia (WM), and one-third of DLBCL^22^. Most primary CLL cells overexpress BCL-2 not only due to the hypomethylation of the BCL-2 gene but also as a result of loss of miR-15 and miR-16 located at 13q14, a region deleted or inactivated by mutations in ~70% of CLL^23^. Chromosomal translocation *t*(14;18)(q32;q21) in 80–90% of FL patients results in constitutive expression of BCL-2 ^24^. Somatic mutations in *BCL-2* in FL are associated with transformation of this indolent disease to a more aggressive DLBCL and decreased patient survival. Some of these mutations increase the affinity of BCL-2 to proapoptotic BH3-only proteins^25^. About one-third of DLBCL also harbor *BCL-2* translocations. Increased *BCL-2* expression has been linked to reduced survival of DLBCL patients. Germinal center B-cell-like (GCB)-DLBCL have increased levels of BCL-2 as a result of the *t*(14;18) translocation, with amplification of the *BCL-2* gene being more often observed in activated B-cell-like (ABC)-DLBCL^26,27^. As a consequence, elevated levels of *BCL-2* are observed in both DLBCL subtypes. Nevertheless, BCL-2 is more abundantly expressed in the ABC- than the GCB-subtype^28^. Besides *BCL-2* translocation and amplification, *BCL-2* gene rearrangement is reported in double-hit lymphomas, which represent ~10% of DLBCL. Additional mechanisms, such as promoter hypomethylation, promoter hypermutation, and phosphorylation, also contribute to its increased expression^29^.

The constitutively active oncogenic transcription factor STAT3 contributes to the malignant phenotype of CLL^30^ and MCL^31^. STAT3 inactivation by the JAK1/2 inhibitor rituximab in NHL resulted in BCL-2 downregulation and sensitization to apoptosis^32^. Additionally, stimulation of the B-cell receptor (BCR) signaling activates JAK2 and STAT3, and JAK1/2 inhibitor ruxolitinib induces apoptosis of CLL cells^30^. BCR signaling is activated and BCL-2 expression increases in Bruton’s tyrosine kinase (BTK) inhibitor ibrutinib-resistant CLL and DLBCL^33,34^. Interestingly, ruxolitinib causes decreased phosphorylation of pSTAT3^Y705^ in chronic ibrutinib-resistant CLL and DLBCL cells, resulting in BCL-2 downregulation and sensitization to venetoclax-induced apoptosis (Kapoor, I et al., unpublished). Aberrant expression of BCL-2 is also found in MCL and MM as a result of cyclin D1 deregulation caused by translocation^35^.

## Establishing BCL-2 family member dependence by BH3 profiling

Cancer cells usually develop a dependency on specific prosurvival BCL-2 proteins due to various factors, such as their tissue of origin, impact of oncogenic lesions driving malignant transformation, and tissue/stromal microenvironment^22,36^. B-cell malignancies, such as CLL and FL, are functionally dependent on BCL-2 for survival, while MM is more dependent on MCL-1 or both MCL-1 and BCL-xL^22^. Determining the functional dependence on select antiapoptotic BCL-2 family proteins has become clinically valuable for patient selection and choice of drug combination. The advent of *“*BH3 profiling*”* using specific BH3-peptides has led the way^37,38^, although more recent availability of specific small-molecule inhibitors of BCL-xL (*Wehi-539A, A1155463, A-1331852*)^39^ and MCL-1 (*A-1210477, S63845*)^40,41^, in addition to BCL-2 inhibition by venetoclax^7^, has facilitated a rapid determination of the BCL-2 family protein dependency in an ex vivo culture. This chemical profiling approach complements the original, elegant, but more laborious peptide-based BH3 profiling pioneered by the Letai group^37,38,42^.

Previous reports have suggested that the ratios between the expression levels of antiapoptotic BCL-2 family members and the proapoptotic BH3-only proteins is correlated with sensitivity to BH3-mimetics. For instance, we showed that the relative expression of (*MCL-1* + *BFL-1*)/*BCL-2* was the most significant predictive marker for the response of primary CLL and leukemic cell lines to ABT-737 treatment^43^. Decreasing the levels of MCL-1 and/or BFL-1 by CDK9-mediated transcriptional inhibition or a targeted genetic approach increased the response to ABT-737 in resistant cells^43^. However, the differential dependence on select antiapoptotic proteins correlates only partly with the relative abundance of the respective proteins. The dependency on BCL-2 or MCL-1 is ultimately assessed by the presence of preformed complexes of BCL-2 with BH3 proapoptotic proteins. An increase in these complexes indicate priming of those cells and increased sensitivity towards specific BH3-mimetics. BCL-2 and MCL-1 are primed with the proapoptotic BH3-only proteins BIM and NOXA, respectively^44^. We previously connected overexpression of MCL-1 and increased complex formation with BIM to ABT-737 resistance. Phosphorylation of MCL-1 at T163 and S64 facilitated not only increased MCL-1 stability but also enhanced binding to BIM following its displacement from BCL-2 and BCL-xL complexes in resistant cells^45^. Additionally, phosphorylation-regulated BCL-2 interactions can also confer resistance to BH3 mimetics^46^, because, apparently, venetoclax cannot bind to phosphorylated BCL-2^47^. Notably, an extensive panel of cell lines has also been engineered to facilitate BH3 profiling by directly assessing the efficacy of BH3-mimetics in preclinical mouse models^44^. These tools, thus, inform selection of the most effective therapeutics to be used rationally based on functional dependence of tumor cells and their ability to induce apoptosis.

## BH3 mimetics as therapeutics

Early attempts to target BCL-2 therapeutically with putative BH3 mimetics, including gossypol compounds and obatoclax, were largely unsuccessful, likely due to a lack of specificity of these drugs^48^. We found that while gossypol was effective in ABT-737-resistant cells, its main action was indirect, by increasing expression of NOXA, which then displaced BIM from MCL-1^45^. To qualify for an authentic, specific BH3 mimetic, a compound must directly and selectively bind the antiapoptotic protein with high-affinity binding and induce MOMP, leading to apoptosis in a BAX/BAK-dependent manner. In addition, it needs to act on the mitochondria, the basis for the peptide-based BH3 profiling^37,38^.

The first potent and selective BCL-2 antagonists were ABT-737, and its orally bioavailable counterpart navitoclax/ABT-263. These *“*BH3 mimetic*”* small molecules bind directly to the BH3-binding domains of antiapoptotic molecules, thereby displacing native BH3-only proteins^49,50^. Navitoclax binds with high affinity to BCL-2, BCL-xL, and BCL-w, but not MCL-1. Initially, it showed antitumor activity in CLL and FL in early phase clinical trials, but further studies were halted because of its on target BCL-xL inhibition in platelets leading to acute thrombocytopenia^51,52^. Exploiting the subtle differences between the binding interfaces of BCL-2 vs BCL-xL led to the development of a highly selective and potent BCL-2 inhibitor, venetoclax. It has a strong affinity for BCL-2, with >100-fold less affinity for BCL-xL or BCL-w^53,54^. By binding to BCL-2, venetoclax disrupts BCL-2 sequestration of BIM, thereby releasing BIM to activate BAX. By acting at the mitochondria, downstream of several other key cell survival pathways, venetoclax is able to induce apoptosis independent of the *TP53* status of the tumor cells^11,54^. The mRNA ratio of (MCL-1 + Bfl-1)/BCL-2 provided a prognostic index marker for the sensitivity of CLL cells to ABT-737, the ratio being lower in the sensitive group as compared to its resistant variants^43^. The ratio of (MCL-1 + *phospho*-BCL-2)/BCL-2 was also found a predictive marker for the response of CLL cells to ABT-199^46^. The sensitivity of cells to ABT-199 in FL was predicted by the BCL-2/BIM ratio^55^, while in MCL by the BCL-2/(BCL-xL + MCL-1) mRNA ratio^56^. Overall, sensitivity to venetoclax is closely associated with high BCL-2 and low BCL-xL or MCL-1 expression levels in MM^57^.

In preclinical models, venetoclax exhibited efficacy against a wide variety of tumor types with no significant thrombocytopenia observed in the in vivo models. Initially approved by the FDA for the treatment of relapsed/ refractory CLL with 17p deletion, it has subsequently gained broad approval including for frontline and relapsed treatment in combination with anti-CD20 monoclonal antibody therapy^8,58^. Additionally, venetoclax as monotherapy demonstrated robust activity in CLL patients who progressed during or after treatment with inhibitors of BTK^59^ and PI3Kδ^60^. In varied histologic subgroups of NHL, the highest overall response rate to venetoclax was in WM and MCL. Responses were less frequent in FL and DLBCL^61^.

## Mechanisms of resistance to BCL-2 inhibition: intrinsic vs acquired resistance

### Intrinsic (primary, innate) resistance

Although venetoclax demonstrated impressive clinical outcomes across a variety of B-cell lymphoid malignancies, certain subtypes are characterized by significantly higher response rates than others (e.g., CLL vs DLBCL). This variation in response rates is attributed to the underlying spectrum of intrinsic resistance mediated by numerous intracellular and microenvironmental factors^62^. Clonal shifts and heterogenous complex evolutionary trajectories have been defined across CLL patients relapsing on venetoclax. Intra-tumoral heterogeneity is reported to trigger clonal evolution and subsequent therapeutic resistance^20^. Mutations contributing to resistance likely pre-exist in sub-clones that confer modest growth advantage or access to supportive microenvironmental niches^20,21^. Several epigenetic factors, including DNA methylation, chromatin remodeling and post-translational histone modifications, dynamically regulate the growth rate and response to environmental pressures, which in turn, influence tumor heterogeneity and clonal evolution^63^.

Microenvironmental agonists, such as IL-10, CD40L, and unmethylated DNA that stimulate TLR9 signaling also contribute to intrinsic resistance, as they lead to robust activation of NF*κ*B signaling in CLL and MCL. Activation of this pathway leads to increased expression of BCL-xL and MCL-1 leading to decreased BCL-2 dependence of tumor cells and thus, tips the balance in favor of cell survival in response to chronic exposure to venetoclax^64^. Similarly, genomic aberrations in the *SWI-SNF* chromatin remodeling complex, mutations in *SMARCA4* and *ARID2* and recurrent copy number deletions in *SMARCA2* confers intrinsic resistance to venetoclax in relapsed/refractory MCL as a result of BCL-xL overexpression. Loss of SMARCA4 function in MCL results in loss of chromatin accessibility at the ATF3 locus, leading to reduced expression of ATF3, which is a direct repressor of BCL-xL expression^65^.

### Acquired (secondary) resistance

Despite an initial response to venetoclax, acquired resistance develops in tumors that eventually relapse leading to clinical disease progression. Therefore, understanding the underlying mechanisms of resistance is crucial for effective molecular targeted therapy. In high-risk CLL patients with *TP53* abnormalities ~50% of patients have relapsed after 2 years on venetoclax as a monotherapy^62^. Whole-exome sequencing and methylation profiling in eight CLL patients before and after relapse to venetoclax revealed genomic instability, with copy number alterations in *BRAF, NOTCH1*, *RB1*, *CD274, SF3B1*, and *TP53* ^66^. Although point mutations affecting the BH3-binding pocket of BCL-2 protein, such as G101V^67,68^, D103Y^69^, and F104I^69^, have recently been reported, relapsed clones also exhibit diverse alternative potential resistance mechanisms, including changes in cellular metabolism and mitochondrial homeostasis.

Overexpression and increased oncogenic dependence of tumor cells on alternative antiapoptotic BCL-xL and MCL-1 proteins contribute to resistance to BH3 mimetics, including venetoclax^29^. Genome-scale loss- and gain-of-function genetic modifier screens in a BCL-2 driven lymphoma cell line as well as an integrated expression profile analysis identified overexpression of MCL-1 and sequestration of BIM as an adaptive mechanism of resistance to venetoclax^70^. Although MCL-1 expression is required for normal physiological processes, such as neuronal differentiation^71^, blood cell maturation^72^, and myocardial homeostasis^73^, upregulation of MCL-1 has emerged as a common determinant of venetoclax resistance^74^. Levels of MCL-1 are modulated at the transcriptional, post-transcriptional, or post-translational level. Additional studies of MCL-1 transcription control have tied its expression to multiple transcription factors, such as STAT3^75^, STAT5^76^, HIF1^77^, or E2F1^78^. Previous studies have implicated the role of post-translational modifications of MCL-1 in the in vitro drug resistance in leukemic B cells^79^. Site-specific mutagenesis revealed that phosphorylation of MCL-1 residues on its PEST domain are responsible for enhancing MCL-1 stability (T92, T163) leading to increased MCL-1 expression, with S64 enhancing BIM binding, both contributing to ABT-737 resistance^45,80^. FL cells resistant to venetoclax showed elevated levels of pERK and pBIM (S69) as well as decreased levels of total BIM^55^. Inhibition of ERK activity eliminated BIM phosphorylation and potentiated venetoclax-induced apoptosis. Importantly, chronic exposure of DLBCL or FL cells to venetoclax results in acquired resistance due to AKT activation and upregulation of MCL-1 and BCL-xL levels that sequestered BIM^55,81^. Downregulation of miR-377 increased BCL-xL expression, thus promoting venetoclax resistance in DLBCL and primary cells from CLL patients^82^.

Metabolites, such as glucose and glutamine, have been shown to also regulate BCL-2, MCL-1, BCL-xL, PUMA, NOXA, and BIM expression and/ or their interactions^83–85^. Therefore, it is not surprising that metabolic changes can alter dependence and response to BH3-mimetics. Glutamine deprivation increases expression and binding of BIM to BCL-2^86,87^, thereby sensitizing MM cells to venetoclax, while metabolic supplementation with α-ketoglutarate reverses this sensitivity^87^. Moreover, reduced mitochondrial respiration, low electron transport chain (ETC) complex I and complex II activities were a predictor and target for venetoclax sensitivity in MM. Inhibition of ETC complex increased BCL-2 dependence and priming to apoptosis via the ATF4-BIM/NOXA axis, thereby sensitizing resistant cells to venetoclax^88^. Reprogramming of mitochondrial outer membrane biology can also lead to changes in the expression of BCL-2 family and increased rates of oxidative phosphorylation, higher rates of reactive oxygen species and MMP in venetoclax-resistant cells in contrast to their sensitive variants^70^. Therefore, interest in targeting metabolic adaptation and deregulated energy metabolism holds promise following the emergence of links between the changes in these pathways and venetoclax resistance.

## Acquiring BCL-2 dependence and vulnerability to venetoclax

CLL cells are sensitized to venetoclax by increasing mitochondrial dependence on BCL-2^89^. BTK inhibition can achieve this in CLL cells by increasing BIM levels and decreasing the abundance or function of MCL-1^89^. Addition of venetoclax to ibrutinib monotherapy resulted in a rapid conversion of partial response to ibrutinib monotherapy into complete response and a steady increase in the proportion of patients with undetectable minimum residual disease in the bone marrow^90^. In a phase II clinical trial of relapsed/refractory MCL patients, ibrutinib followed by addition of venetoclax resulted in improved clinical outcomes^91^. In addition, while initially resistant to venetoclax, malignant B cells that no longer respond to BTK inhibition become instead responsive to BCL-2 inhibition. We found that acquiring sensitivity to venetoclax is based on gaining BCL-2 dependence, partly based on STAT3-dependent increased BCL-2 expression in chronic ibrutinib-resistant CLL and DLBCL cells (Kapoor, I et al., unpublished). Moreover, ibrutinib reduces the expression of the chemokine receptor CXCR4 and thereby homing of the malignant B cells to the supportive microenvironment that affects sensitivity to BCL-2 inhibition^56^. Together, these data provide a rationale to support the combination of venetoclax with BTK inhibitors (ibrutinib, acalabrutinib) for treating venetoclax-resistant CLL. Further, in a phase I/II study of relapsed/refractory CLL patients, combination of umbralisib, a novel highly specific PI3Kδ inhibitor and ublituximab, a chimeric anti-CD20 monoclonal antibody with venetoclax demonstrated good tolerability with undetectable minimum residual disease, representing an effective treatment strategy for relapsed/refractory CLL patients^92^. Taken together, these data provide a rationale for combination of venetoclax with novel agents to reprogram apoptotic dependencies in CLL for treating venetoclax-resistant CLL with deeper prolonged remissions and defined treatment durations.

## Targeting resistance to BCL-2 inhibition

Based on the complex interplay between BCL-2 family proteins and the diverse resistance mechanisms discussed above, therapeutic strategies using venetoclax as monotherapy may lead to eventual treatment failure with the potential for progression to a more aggressive disease. In contrast, a rational combination of drugs to target multiple driver genes simultaneously is promising, not only for inhibiting more clones in a tumor but also making new cancer-promoting mutations more difficult to be selected and propagated^93^. Thus, there is a clear clinical need to identify novel treatment strategies for patients with relapsed/refractory aggressive B-cell malignancies. Targeted therapy directed against epigenetic modifiers, alternative antiapoptotic BCL-2 proteins, cellular metabolism/energetics, and compensatory prosurvival pathways offers exciting opportunities for achieving durable remissions.

## Targeting epigenetic modifications

With emerging knowledge of mutational landscapes in B-cell malignancies through high-throughput sequencing, it has become apparent that different epigenetic deregulations play a crucial role in driving tumorigenesis. Mechanisms for these epigenetic alterations include DNA methylation, histone modifications, and chromatin remodeling^94^. Additionally, genetic mutations of epigenetic modifier enzymes, and histone proteins, further expand the heterogeneity of epigenetic modifications in B-cell malignancies^95^. For instance, activating mutations in *EZH2*, a histone methyltransferase gene, were found in ~22% of GCB-DLBCL and ~7% of FL patients^96^. Loss-of-function mutations were observed in *KMT2D* (~90% of FL and ~30% of DLBCL)^97,98^, *CREBP*, or the *EP300* that have been reported in 40% of DLBCL and FL patients^97^. Recurrent point mutations in *MEF2B* have been described in ~15% of FL and ~13% GCB-DLBCL^97^. Together, the combination of different chromatin alterations changes positioning of nucleosomes, packaging of DNA, chromatin accessibility to transcriptional regulators, thus modulating gene expression^94^. Based on widespread epigenetic dysregulations and the dynamic nature of these alterations, therapeutic intervention with epigenetic drugs combinations seems promising. Thus, combination of the pan-HDAC inhibitor, panobinostat and MEK inhibitor or venetoclax effectively induces apoptosis in MM cells. Mechanistically, the MEK inhibitor increases BIM levels, while panobinostat acts as a de facto MCL-1 and BCL-xL inhibitor, dissociating BIM:MCL-1 and BIM:BCL-xL complexes in MM cells. Moreover, venetoclax exposure further increases release of BIM from BCL-2, leading to increased expression of BIM:BAK and BIM:BAX complexes resulting in synergistic induction of apoptosis^99^. Further, in a phase I, open-label, dose-escalation study, combination of fimepinostat (CUDC-907), a PI3K and HDAC inhibitor, with venetoclax and/or rituximab has been tested for antileukemic activity and tolerability in patients with relapsed and/or refractory DLBCL or high-grade B-cell lymphoma with or without MYC and BCL-2 alterations (NCT01742988).

Therefore, an understanding of the link between epigenetic defects and cancer is clinically invaluable for predicting disease prognosis and treatment. The combinations of epigenetic therapies with targeted anticancer agents potentiate the responses to existing therapies in the treatment and prevention of malignant transformation.

## Targeting MCL-1

**Fig. 2:**
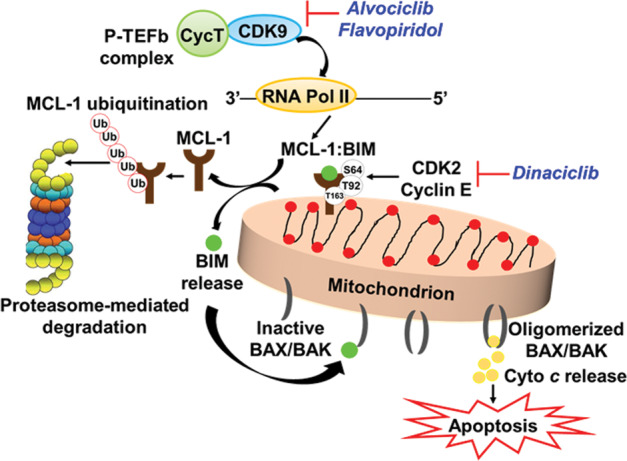
Role and regulation of MCL-1-mediated resistance. Transcriptional control is achieved by CDK9 associated with Cyclin T1 (CycT) to form the positive transcription elongation factor b (P-TEFb) complex. Inhibition of CDK9 reduces MCL-1 expression via blocking RNA polymerase II-mediated transcriptional elongation. Alternatively, Cyclin E/CDK2 inhibition can destabilize MCL-1 by inhibiting its phosphorylation on: (i) PEST domain residues (T92, T163) resulting in MCL-1 ubiquitination and proteasome-mediated degradation and (ii) on residue S64 that changes binding affinity to BIM, thus releasing BIM from its interaction with MCL-1 and causing BAX/BAK activation and apoptosis.

Although MCL-1 plays a role in normal physiological processes, including cardiac and hepatic tissues, overexpression of MCL-1 and increased oncogenic addiction of tumor cells has emerged as a common determinant of venetoclax resistance^100^. Therefore, MCL-1 inhibition is an attractive therapeutic target in lymphoid malignancies that depend on its prosurvival activity. For instance, in aggressive MCL, MCL-1 inhibition, both alone or together with venetoclax, ibrutinib, or BCL-xL inhibition synergistically induced apoptosis and effectively eradicated lymphoma cells protected by the microenvironment^101^. Targeting MCL-1 can be achieved by agents that bind to and inactivate MCL-1 and disrupt high-affinity protein−protein interactions, or disrupt MCL-1 stability (Fig. 2)^100^. S63845 was the first highly potent, selective inhibitor that specifically binds with high affinity to the BH3-binding groove of MCL-1. S63845 is ~1000-fold more potent in killing MCL-1-dependent MM cells than A-1210477; both bind to MCL-1 selectively and disrupt MCL-1-BIM complexes in living cells^41,102^. As a single agent, S63845 is effective in MM, leukemia, and lymphoma cells, by interfering with BAK and BAX binding to MCL-1, while sparing normal tissues at efficacious doses^41^. S63845 in combination with venetoclax synergized in vivo in relapsed MCL and induced synthetic lethality by concurrent inhibition of MCL-1 and BCL-2^103^. Additionally, other selective MCL-1 inhibitors recently developed, AZD5991^104^, AMG 176^105^ synergize with venetoclax and induce apoptosis in MM.

Although venetoclax has potent antileukemic efficacy, resistance can occur due to its inability to inhibit MCL-1, which is stabilized by the MAPK pathway^106^.The RAS/BRAF/MAPK/ERK cascade is activated by mutations in novel CLL drivers, such as *NRAS*, *KRAS*, *BRAF*, *PTPN11*, and *MAP2K1/2* (MEK1/2)^107^. Expression of MCL-1 is regulated by the MAPK at multiple levels. ERK1/2 has been shown to promote *MCL-1* gene expression by ELK1 and stabilizes protein expression by phosphorylation of its PEST domain^108^. Thus, a strategy to suppress MCL-1 expression by targeting MAPK should sensitize malignant cells to BH3 mimetics, including venetoclax. The MEK1/2 inhibitor binimetinib potentiated the activity of venetoclax and ABT-737 under conditions that mimic the CLL tumor microenvironment via downregulation of MCL-1 activity, BIM and BCL-xL expression^109^.

**Fig. 3:**
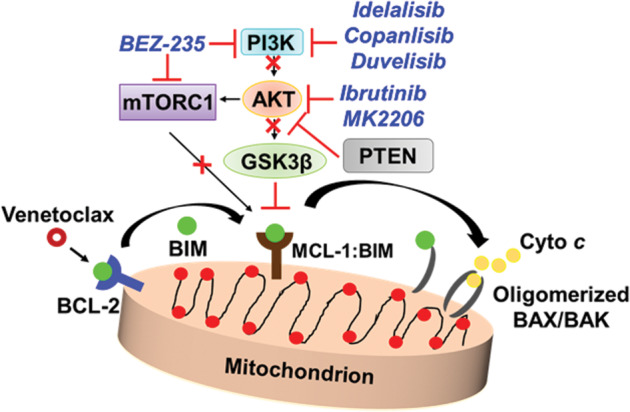
Targeting a compensatory prosurvival pathway in venetoclax resistance. The dual PI3K/mTOR inhibitor NVP-BEZ235 inhibits the PI3K and the mTOR pathway, which interferes with MCL-1 stability, thereby releasing BIM to activate BAX/BAK, leading to cytochrome *c* release and apoptosis. Inhibition of PI3Kδ by idelalisib and AKT by ibrutinib and MK2206 further blocks the PI3K/AKT-mediated signaling. Inhibition of these pathways reduces MCL-1 levels, leading to BIM release and apoptosis.

Inhibition of the PI3K/AKT/mTOR pathway has been also shown to be an attractive strategy to overcome venetoclax resistance indirectly by reducing MCL-1 levels^110^ (Fig. 3). siRNA-mediated downregulation of both AKT and MCL-1-sensitized venetoclax-resistant DLBCL cells to apoptosis. Cotreatment with the PI3Kδ inhibitor idelalisib and venetoclax decreased MCL-1 expression and increased apoptosis in association with AKT-mediated BAX activation^81^. Subsequently, others confirmed synergy between venetoclax and the second-generation PI3K inhibitors, copanlisib^111,112^ and duvelisib^113^ in malignant hematopoietic cells through MCL-1 downregulation (Fig. 3).

Inhibition of CDK2/Cyclin E-mediated MCL-1 phosphorylation by low concentration of dinaciclib-sensitized ABT-737-resistant DLBCL cells to apoptosis by promoting release of sequestered BIM from MCL-1. Strikingly, dinaciclib in combination with venetoclax resulted in robust synergistic induction of apoptosis in DLBCL and primary CLL cells^80^. Although higher concentrations of dinaciclib decrease MCL-1 mRNA levels by blocking CDK9-mediated Pol II transcription, inhibition of MCL-1 by dinaciclib amplified venetoclax-induced apoptosis in DLBCL^114^ (Fig. 2). Importantly, antitumor activities of these combinations have also shown encouraging results in xenografts and in a genetic murine model of Myc-BCL-2 double-hit lymphoma^115^. In conclusion, inhibition of MCL-1 either through direct or indirect agents in conjunction with a BH3-mimetic therapy emerges as the most effective means to overcome venetoclax resistance by selectively eradicating malignant cells that depend on MCL-1 for its prosurvival activity.

## Targeting metabolic vulnerabilities and cellular energetics

The metabolic properties of cancer cells are markedly distinct from those of normal cells. Mounting evidence shows that many metabolic features characteristic to cancer cells, such as dysregulated Warburg-like glucose metabolism, increased fatty acid synthesis, glutaminolysis, increased mitochondrial metabolism and less dependence on OXPHOS at least, in part, drive chemoresistance in cancer and thus have crucial implications for targeted and efficacious chemotherapies^116–118^. Therefore, by targeting the metabolic pathways that import, catabolize, and synthesize essential cellular components, drug-resistant cancer cells can often be effectively resensitized to apoptosis^118^.

The uptake of glucose and glutamine is often increased through upregulation of transporter proteins, such as the GLUT family glucose transporter or the ASC2 amino acid transporter. Therefore, targeting these proteins has recently become a major focus of interest. For instance, MM cells are reliant on glucose and glutamine and withdrawal of either nutrient is associated with varying levels of apoptosis^117^. Inhibition of glucose metabolism with the GLUT4 inhibitor ritonavir blocks proliferation in MM and induces apoptosis by reducing MCL-1 levels that is enhanced with venetoclax, suggesting that nutrient deprivation may re-configure BCL-2 family protein dependence in MM cells and enable sensitization to venetoclax^119^. Additionally, hexokinase inhibitors, such as 2-deoxyglucose (2-DG) and lonidamine, enhance ABT-263/737-induced apoptosis both in vitro and in vivo^83,120^. 2-DG induces apoptosis by reducing MCL-1 levels indirectly by inhibition of glycolysis and depleting ATP levels, leading to activation of AMPK and inhibition of MCL-1 translation^83,120^. Additionally, it disrupts the interaction between BAK and MCL-1, thereby increasing the ability of ABT-263/737 to release BAK from the MCL-1/BCL-xL/BAK^121^. Targeting glutamine metabolism in MM-sensitized MM cells and primary relapsed/refractory patient samples to venetoclax by inducing metabolic shift and increasing expression and binding of BIM to BCL-2 ^87^. Therefore, targeting metabolic reprogramming in resistant cells in the majority of tumors represents a promising therapeutic avenue to circumvent chemoresistance and residual diseases.

## Summary

**Fig. 4:**
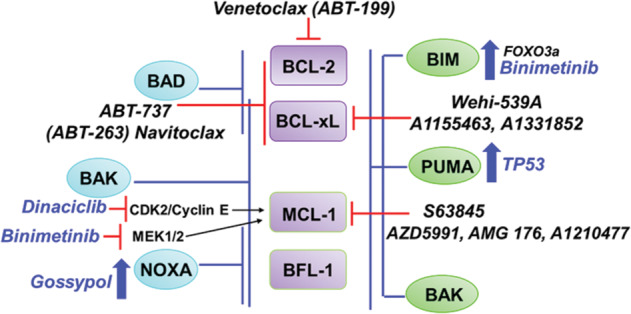
BCL-2 family proteins: overcoming blocks in apoptosis. Schematic illustration of the interaction between pro- and antiapoptotic proteins, and small-molecule inhibitors that impede these interactions to overcome apoptosis block. The antiapoptotic proteins are shown in the center (purple). Proapoptotic proteins that bind to all of the antiapoptotic proteins are shown on the right (green), while the proapoptotic proteins with preferential binding are shown on the left (blue). Direct inhibition by various BH3 mimetics are depicted in black text, while several indirect means to modulate the proapoptotic proteins are shown in blue text.

BCL-2 family proteins play a critical role in promoting survival of tumor cells. In particular, BCL-2 has a prosurvival role for CLL, DLBCL, and MM and therefore, it represents a rational therapeutic target (Fig. 4). Venetoclax, which selectively inhibits BCL-2, is most effective clinically in CLL. Yet, despite impressive clinical outcomes, venetoclax monotherapy may not maximize therapeutic efficacy, and de novo or acquired drug resistance invariably emerge. With growing mechanistic evidence of clonal shifts, a complex interplay of BCL-2 family proteins, metabolic reprogramming or tumor microenvironment in B-cell malignancies, future studies should continue to define biomarkers that could predict the response of venetoclax. As the field advances, studies investigating the underlying mechanisms of modulating cancer cell dependency, upregulating proapoptotic proteins, and modulating cellular energy metabolism will continue to optimize the effectiveness of venetoclax. This may also open avenues for strategic combinations that could be leveraged to overcome resistance mechanisms that have been identified with venetoclax monotherapy. The increasing number of therapeutic options, in conjunction with advancing molecular testing, such as BH3 profiling may enable dynamic, personalized modifications to treatment for durable disease remission.
